# Supplementary material for: Vegetation-based climate mitigation in a warmer and greener World
Source: Nat Commun. 2022 Feb 1;13:606. doi: 10.1038/s41467-022-28305-9 (PMC8807606; doi:10.1038/s41467-022-28305-9)
Supplement: Supplementary file 1 — Supplementary Information [file 41467_2022_28305_MOESM1_ESM.pdf]

Supplementary figures

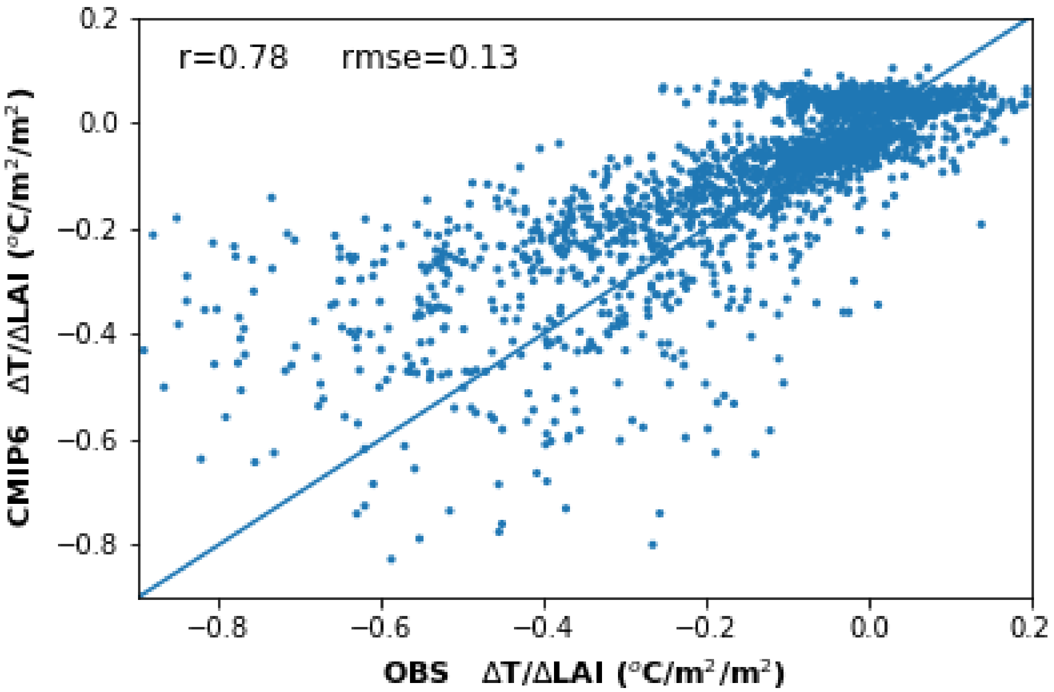

Figure 1 Spatial correlation between the estimated sensitivity of air temperature to LAI derived from Earth observations (x-axis) and CMIP6 simulations (y-axis) over 2003-2014, as reported in Fig 1cd.

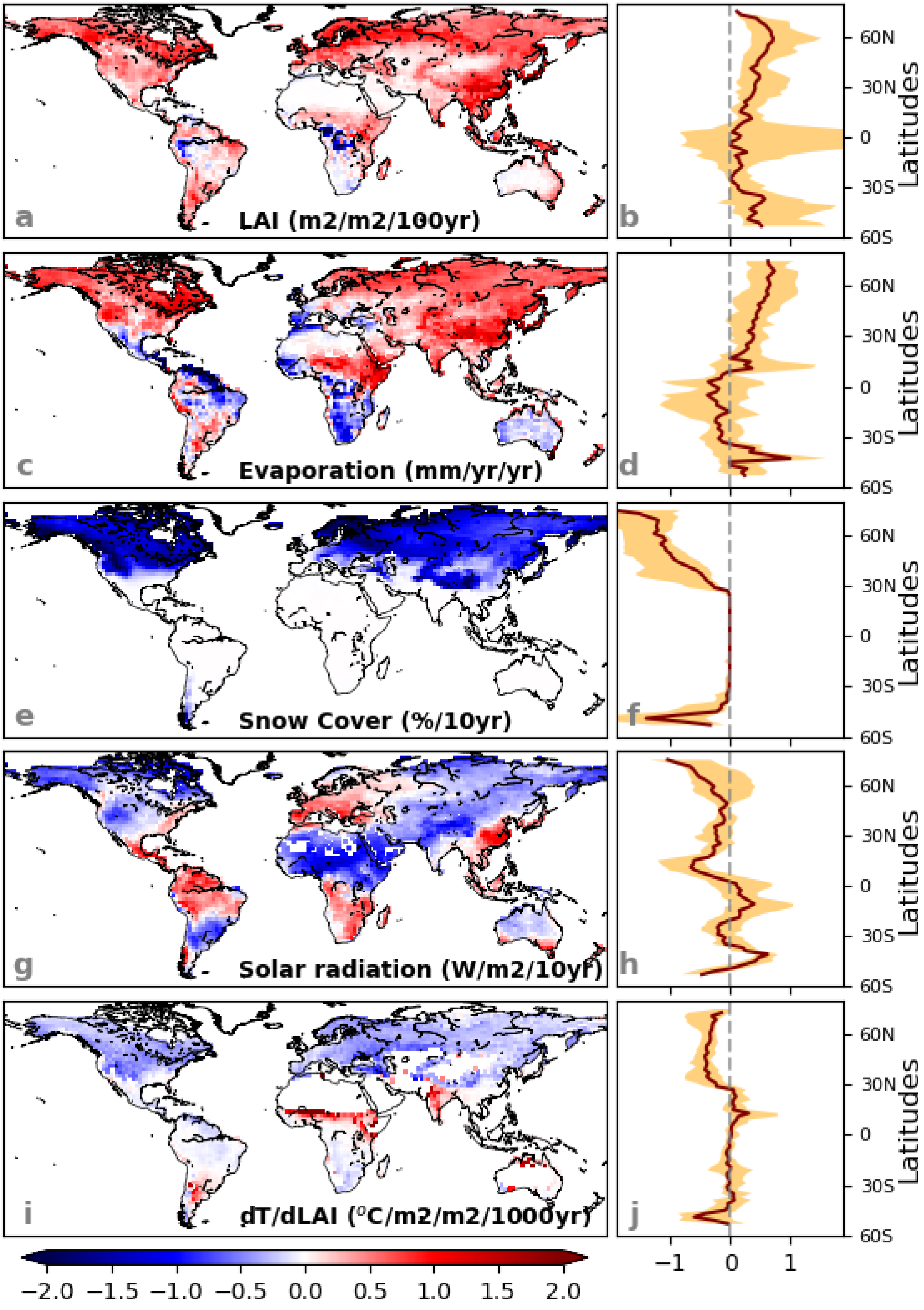

Figure 2 Trends in LAI, evaporation, snow cover averaged over the year, surface downwelling shortwave solar radiation (SWdown) and dT/dLAI from and ensemble of 18 CMIP6 models over 2015-2100 under SSP370 scenario. The median is shown in the left panel (a, c, e, g, i) while the right panels (b, d, f, h, j) shows the median (solid line) and the min and max zonal mean of the model ensemble (orange envelope).

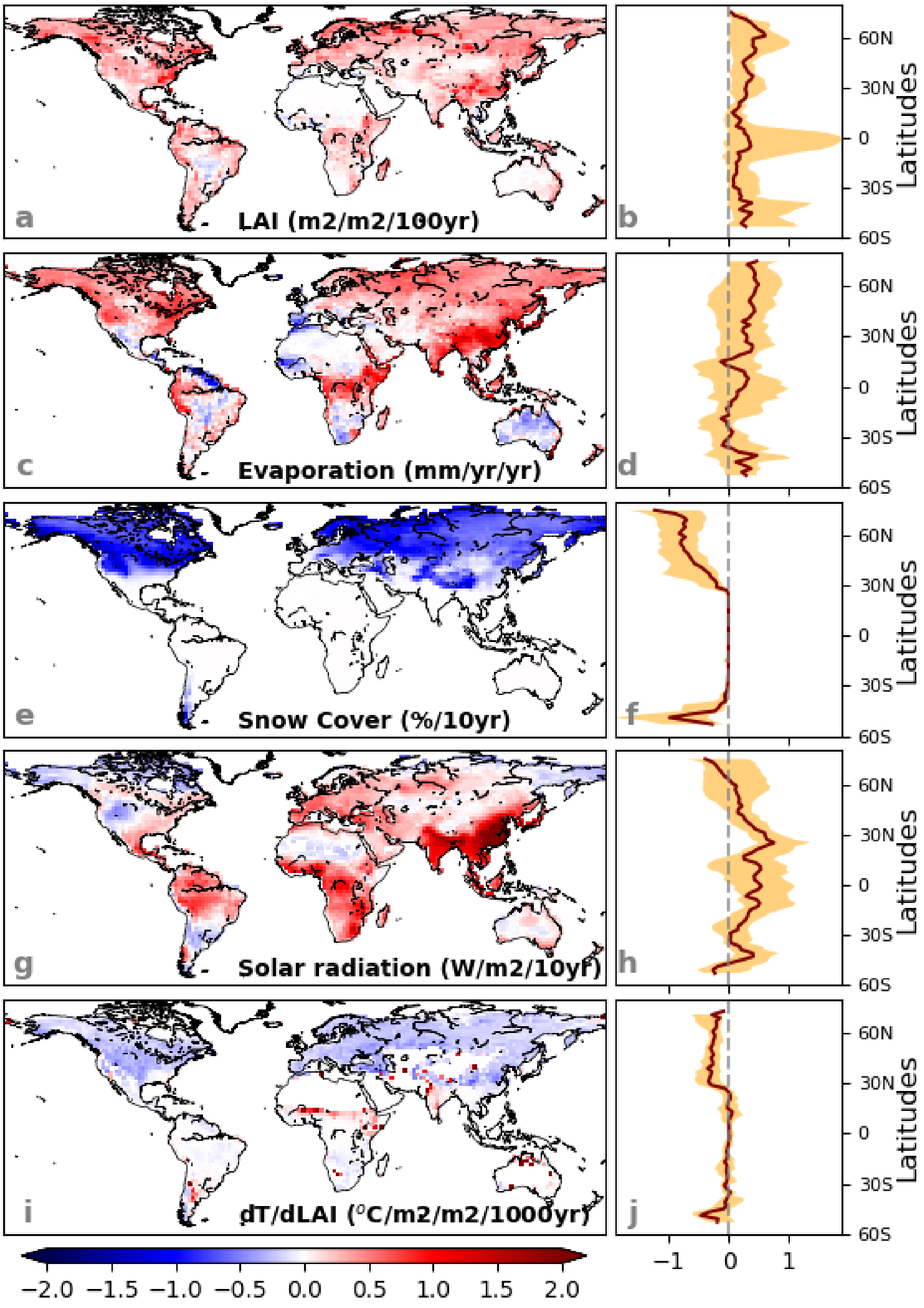

Figure 3 Trends in LAI, evaporation, snow cover averaged over the year, surface downwelling shortwave solar radiation (SWdown) and dT/dLAI from and ensemble of 18 CMIP6 models over 2015-2100 under SSP245 scenario. The median is shown in the left panel (a, c, e, g, i) while the right panels (b, d, f, h, j) shows the median (solid line) and the min and max zonal mean of the model ensemble (orange envelope).

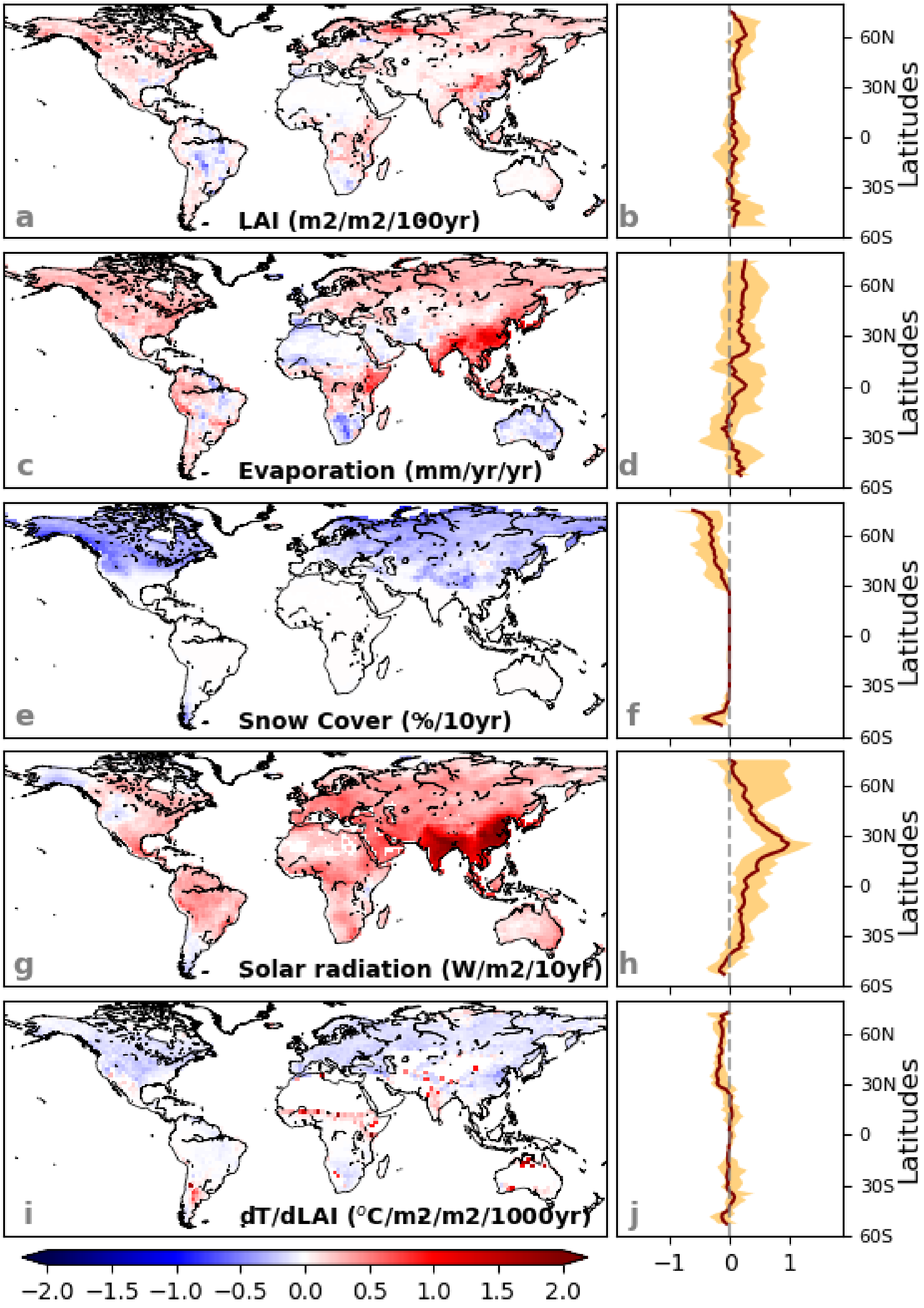

Figure 4 Trends in LAI, evaporation, snow cover averaged over the year, surface downwelling shortwave solar radiation (SWdown) and dT/dLAI from and ensemble of 18 CMIP6 models over 2015-2100 under SSP126 scenario. The median is shown in the left panel (a, c, e, g, i) while the right panels (b, d, f, h, j) shows the median (solid line) and the min and max zonal mean of the model ensemble (orange envelope).

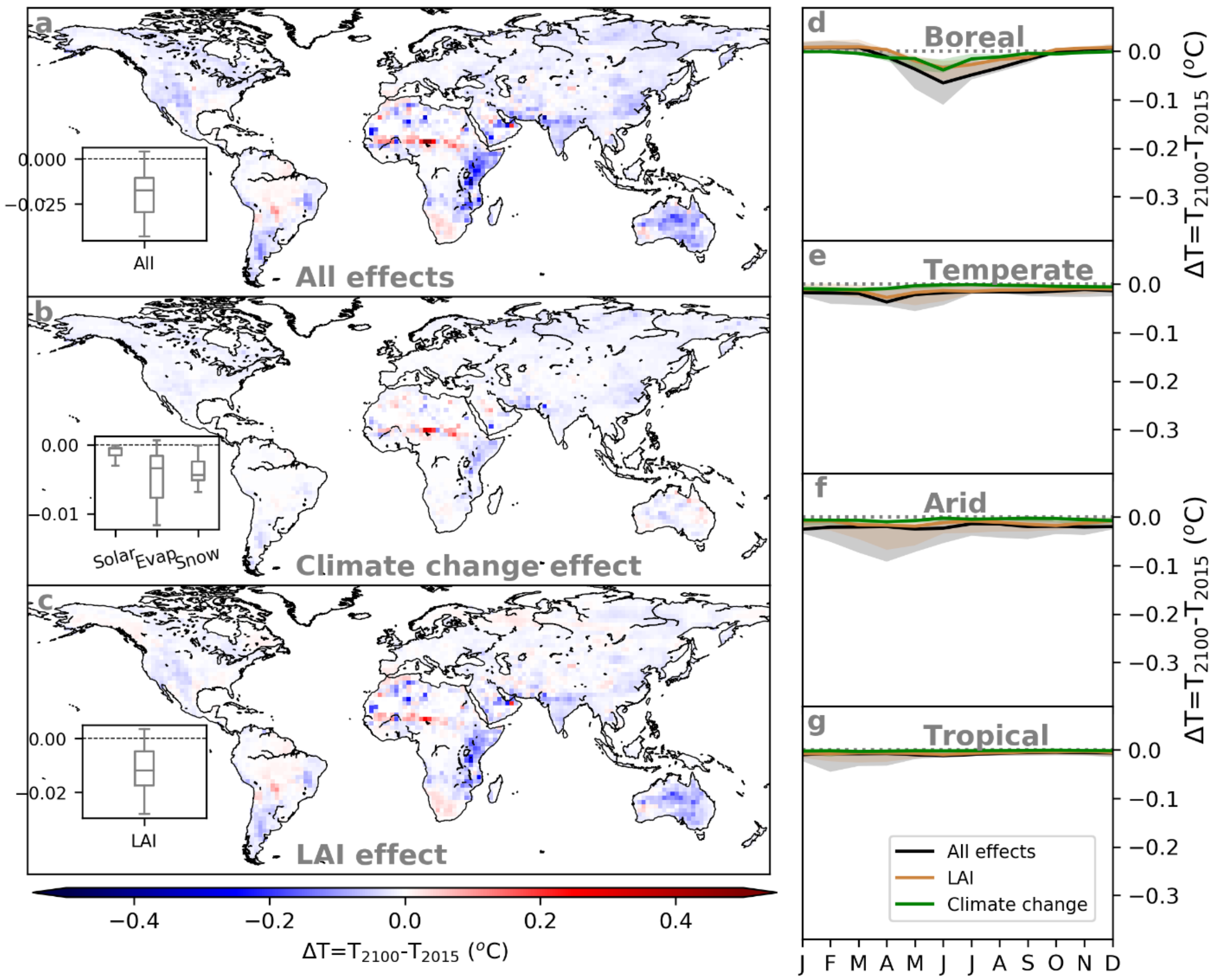

Figure 5 Annual maps and seasonal cycle of temperature change over the four climate zones induced by LAI dynamics over 2015-2100 under SSP126 scenario. Black line is the median of CMIP6 model simulations that accounts for the changing background climate conditions, while the brown line case assumes a constant current climate condition. Grey and brown envelopes come from the CMIP6 models showing min and max temperature change.

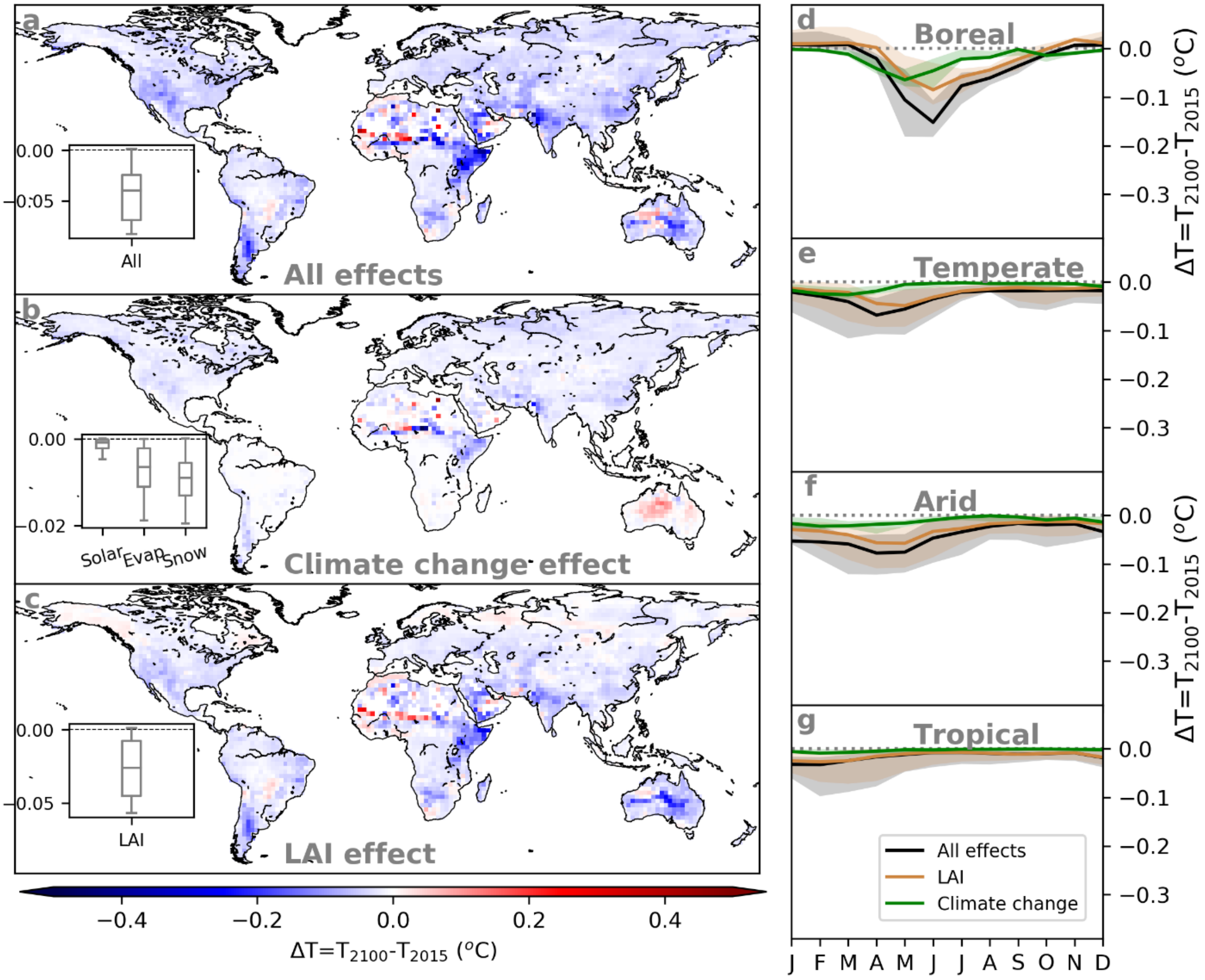

Figure 6 Annual maps and seasonal cycle of temperature change over the four climate zones induced by LAI dynamics over 2015-2100 under SSP245 scenario. Black line is the median of CMIP6 model simulations that accounts for the changing background climate conditions, while the brown line case assumes a constant current climate condition. Grey and brown envelopes come from the CMIP6 models showing min and max temperature change.

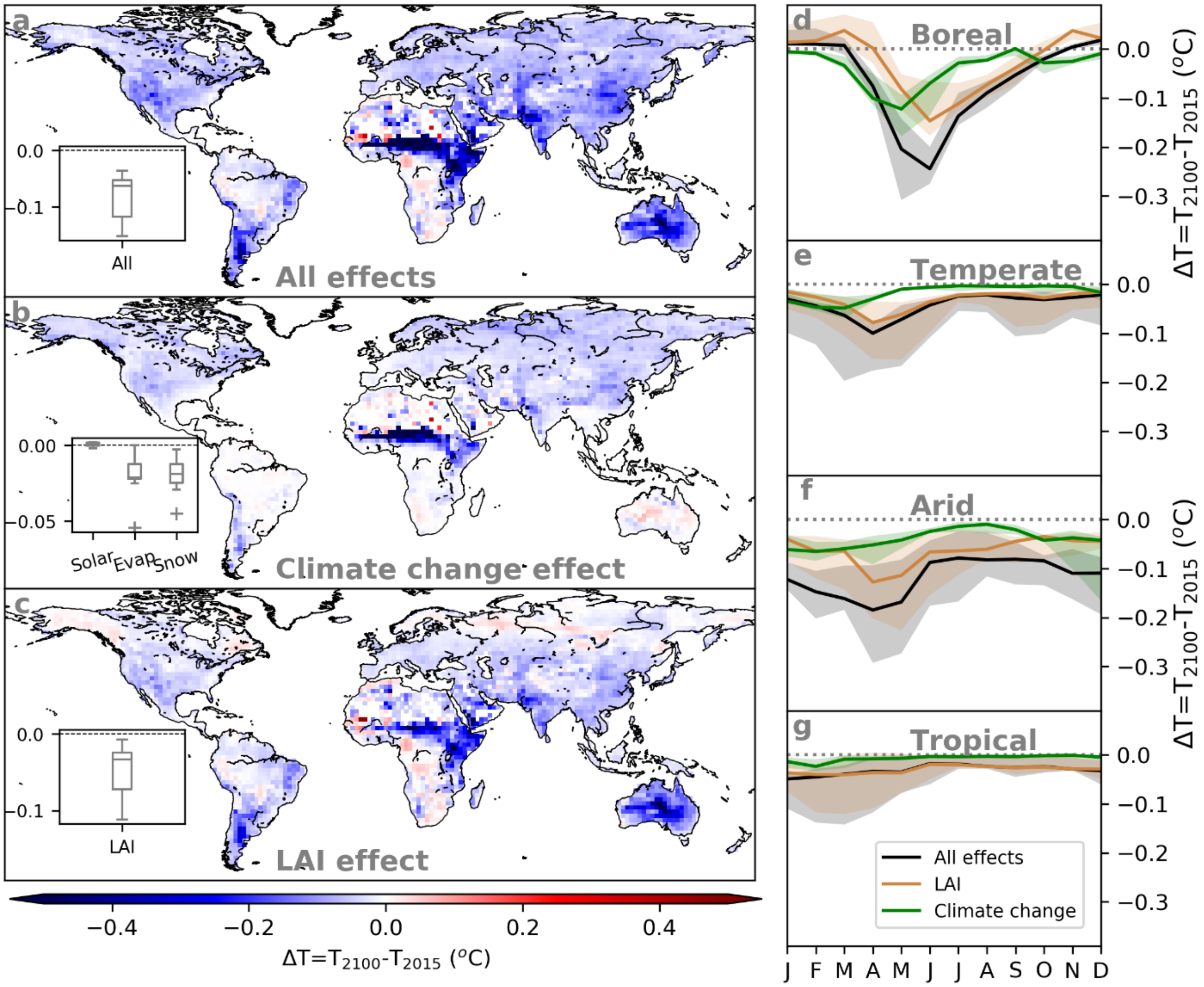

Figure 7 Annual maps and seasonal cycle of temperature change over the four climate zones induced by LAI dynamics over 2015-2100 under SSP370 scenario. Black line is the median of CMIP6 model simulations that accounts for the changing background climate conditions, while the brown line case assumes a constant current climate condition. Grey and brown envelopes come from the CMIP6 models showing min and max temperature change.

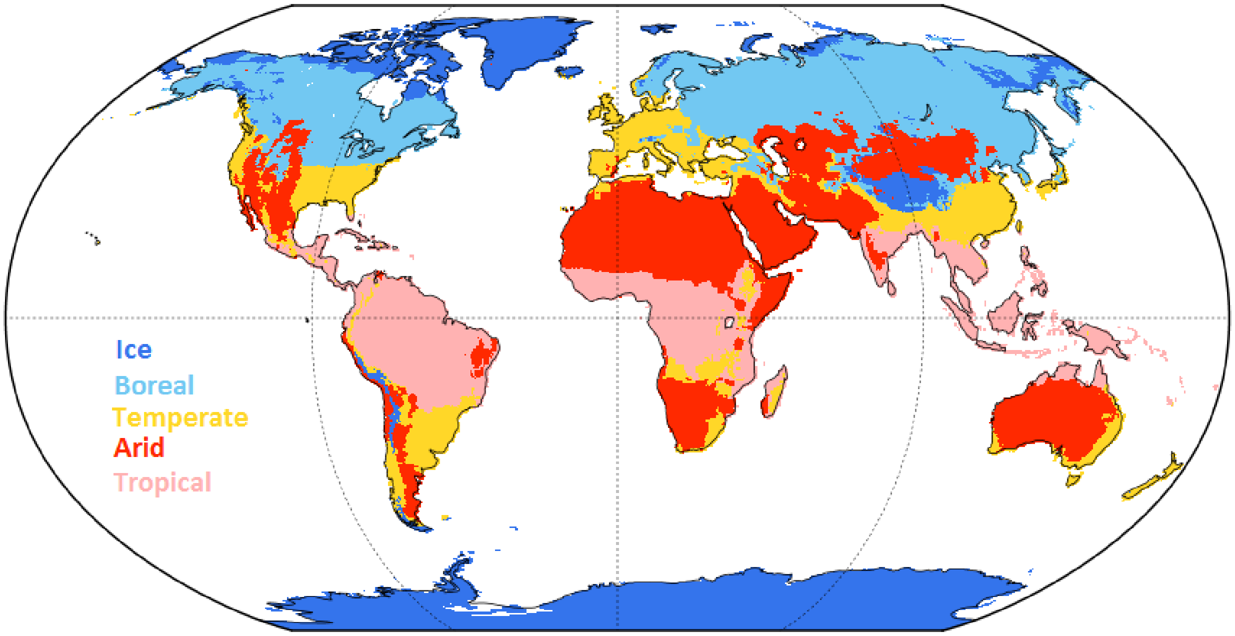

Figure 8 Köppen-Geiger World map of climate classification for the second half of the 20th century.

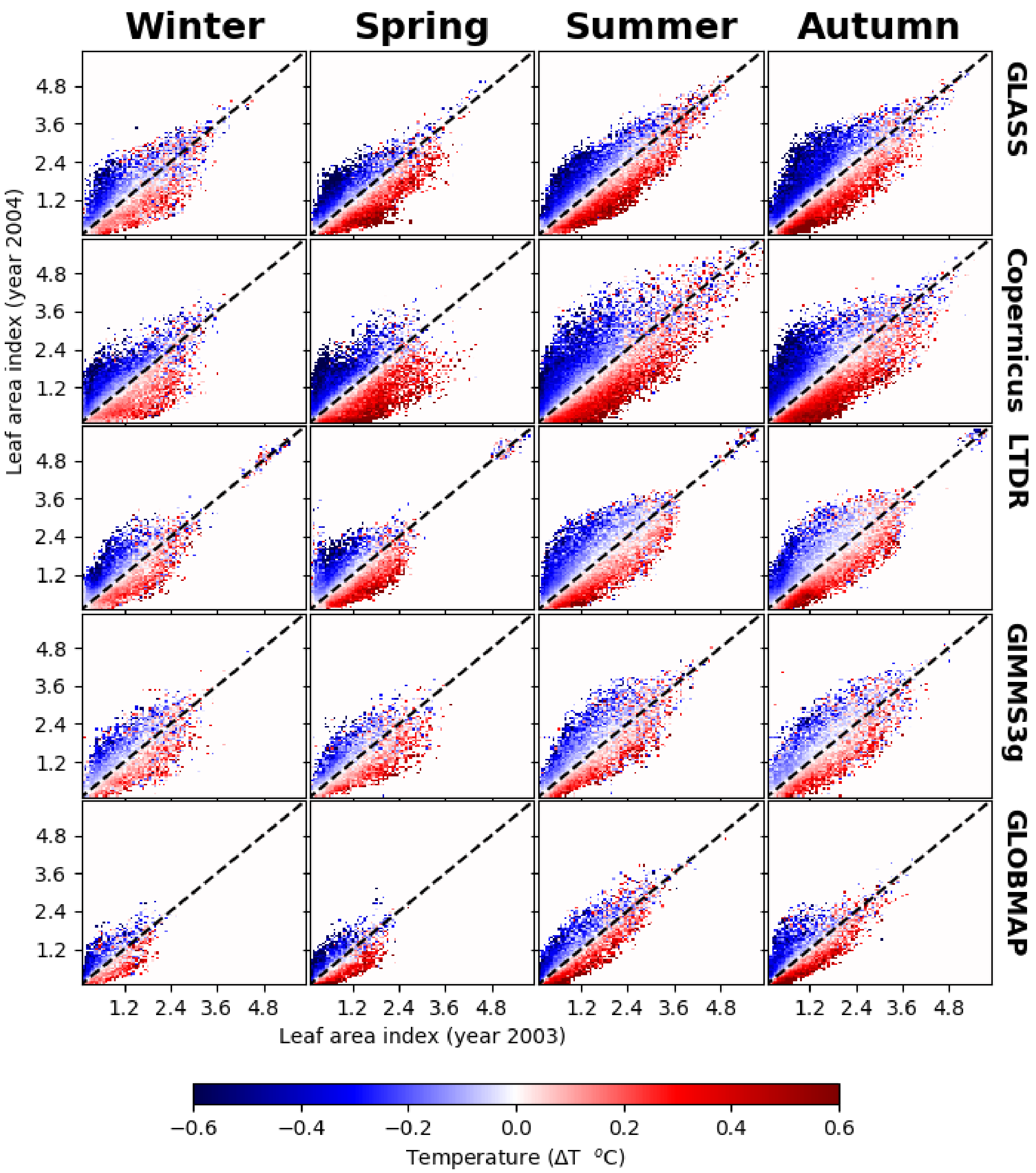

Figure 9 Variations in mean seasonal air surface temperatures due to land cover change between 2003 (X axis) and 2004 (Y axis) over the Arid climate zone.

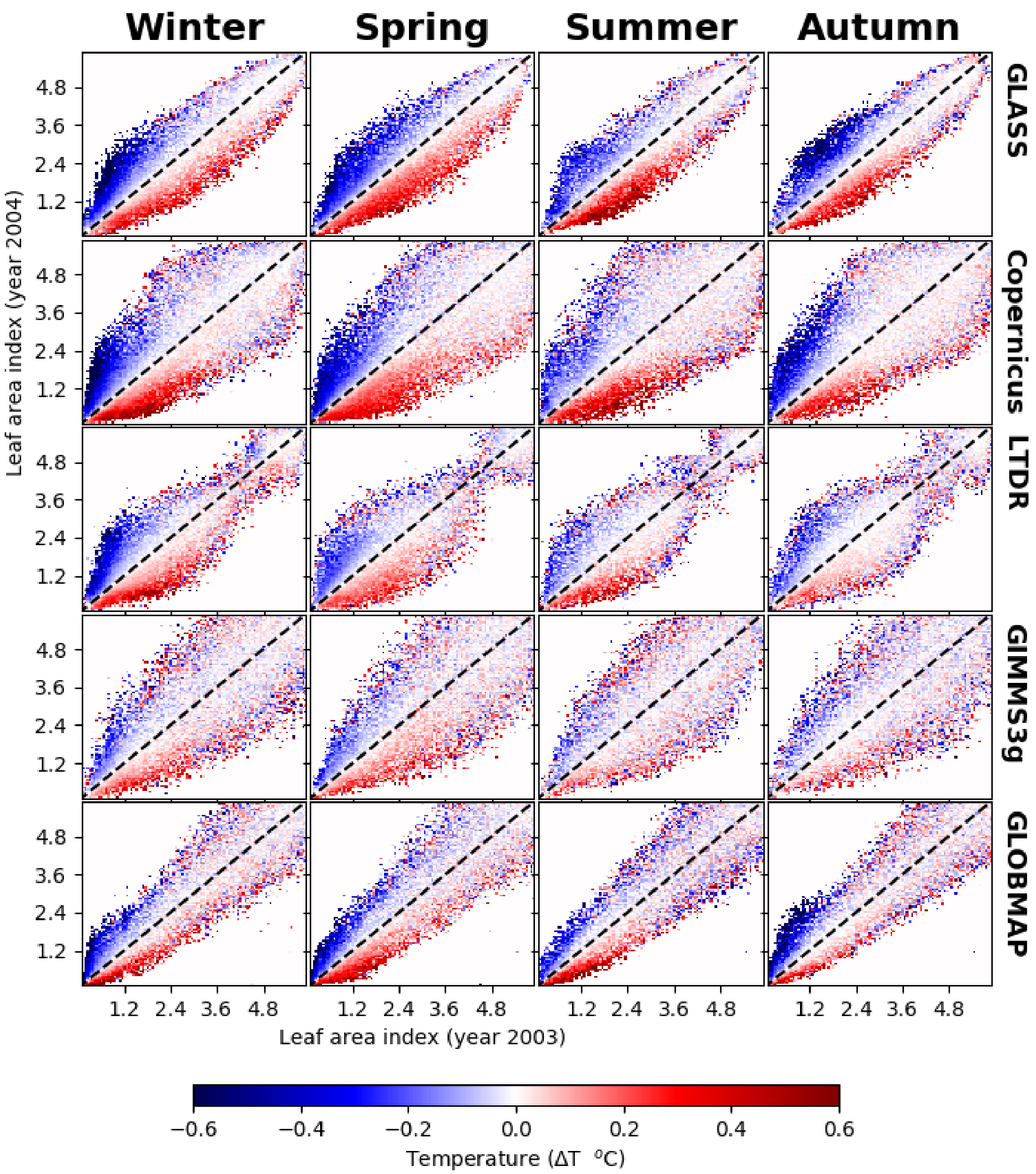

Figure 10 Variations in mean seasonal air surface temperatures due to land cover change between 2003 (X axis) and 2004 (Y axis) over the Tropical climate zone.

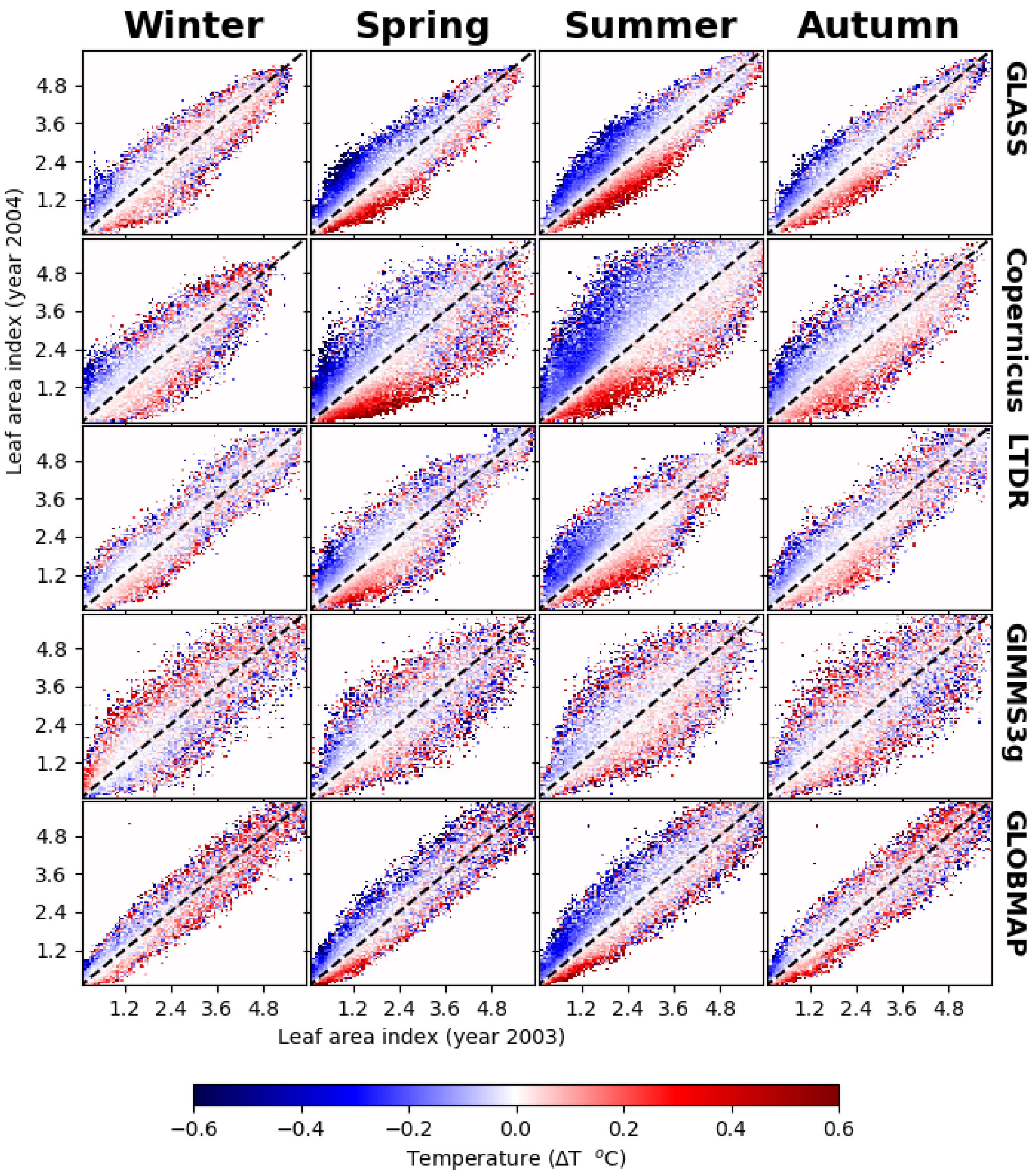

Figure 11 Variations in mean seasonal air surface temperatures due to land cover change between 2003 (X axis) and 2004 (Y axis) over the Temperate climate zone.

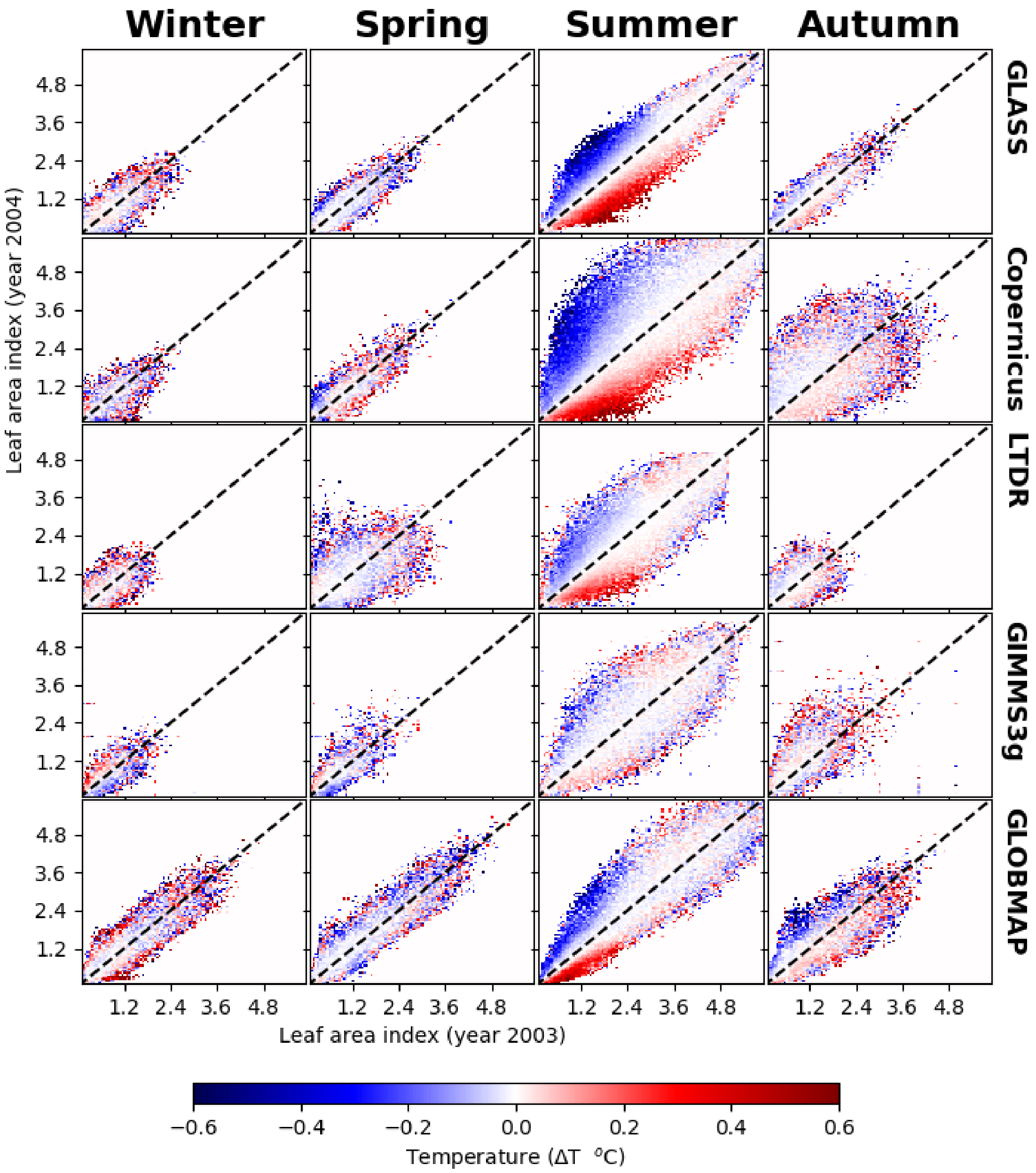

Figure 12 Variations in mean seasonal air surface temperatures due to land cover change between 2003 (X axis) and 2004 (Y axis) over the Boreal climate zone.

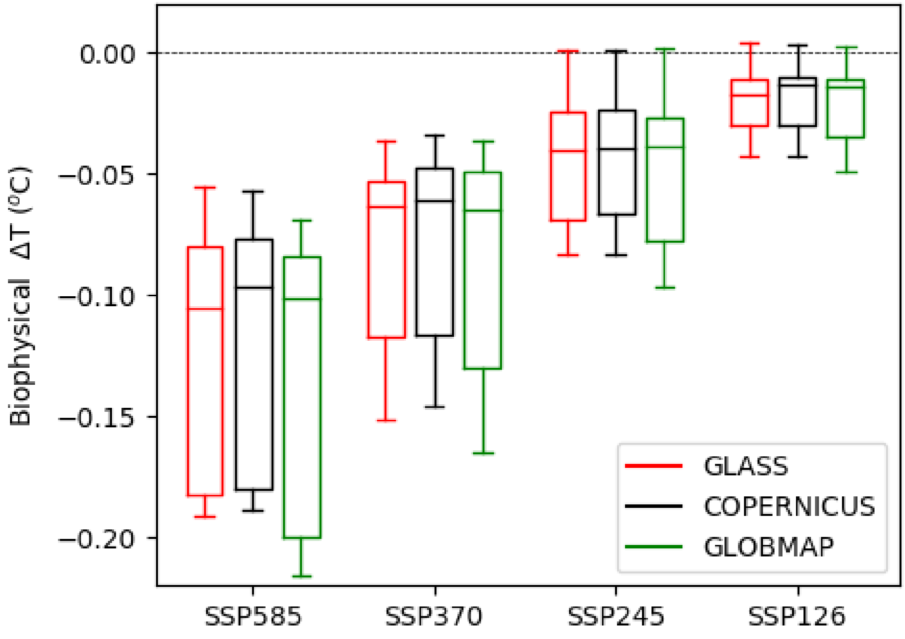

Figure 13 Mean biophysical impact of LAI change on air temperature over 2015-2100 under the four SSPs, based on GLASS LAI in red and COPERNICUS in black and GLOBMAP in green.

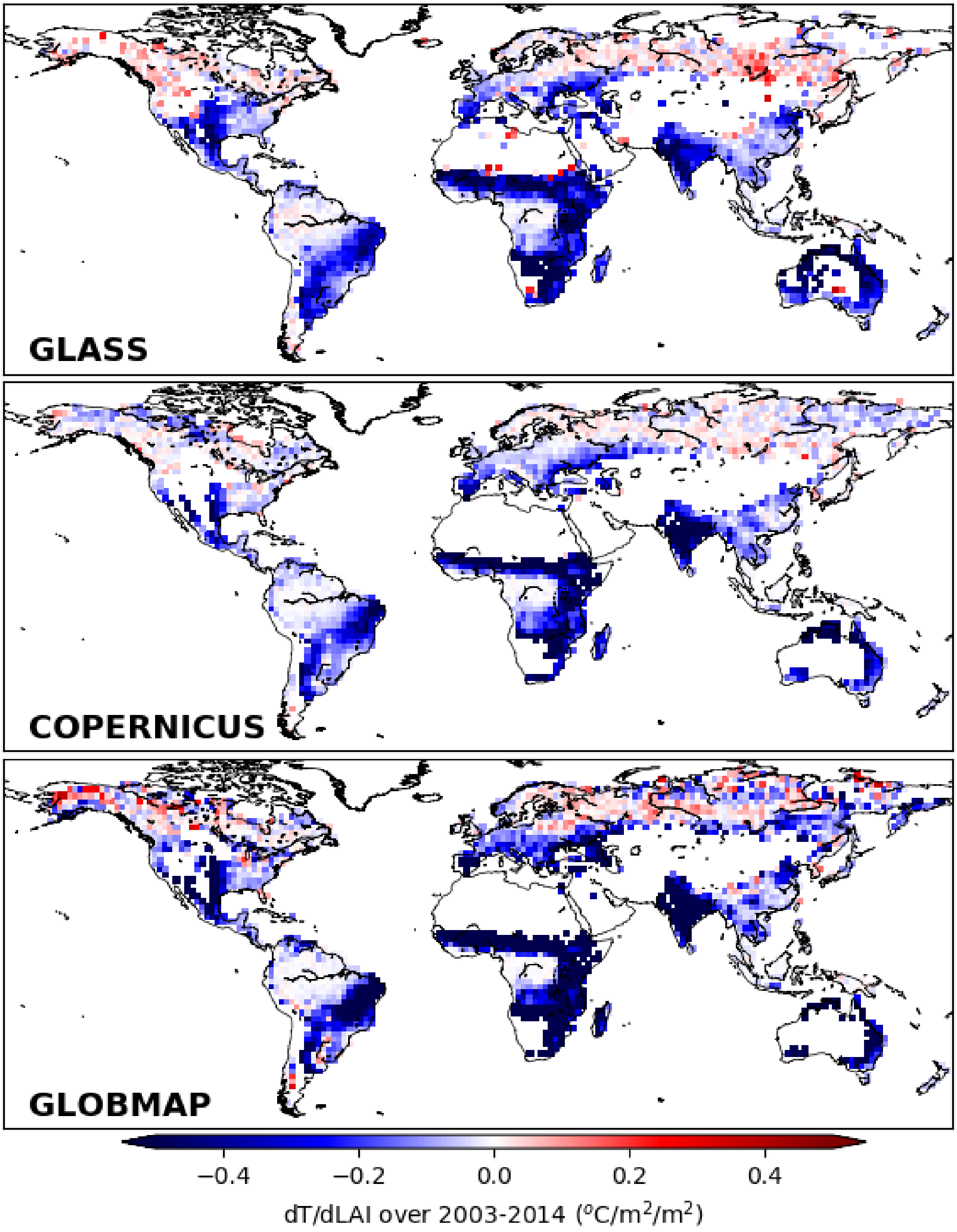

Figure 14 Mean annual sensitivities derived from Earth observations by using all combinations of pairs of years in 2003-2014 for each month of the year using GLASS LAI on top, COPERNICUS in middle and GLOBMAP in bottom.

Supplementary table

Table 1 Mitigation potential of vegetation density change over 2015-2100 for the SSP585. These numbers came from the average of 18 models ± standard deviation.

Biophysical (mean±std)	Biochemical (mean±std)	Total (mean±std)
0.12±0.05	0.59±0.41	0.71±0.40
